# KLF5 promotes esophageal squamous cell cancer through the transcriptional activation of FGFBP1

**DOI:** 10.1007/s12032-023-02244-x

**Published:** 2023-12-12

**Authors:** Fengyun Wang, Ming Luo, Yufeng Cheng

**Affiliations:** 1 Department of Oncology, First Affiliated Hospital of Baotou Medical College, Inner Mongolia University of Science & Technology, Baotou, Inner Mongolia China; 2 Imaging Department, Third Affiliated Hospital of Baotou Medical College, Baotou, Inner Mongolia China; 3 Department of Radiotherapy, Qilu Hospital, Cheeloo College of Medicine, Shandong University, No.107, West Wenhua Road, Lixia District, Jinan, Shandong China

**Keywords:** KLF5, FGF-BP1, Metastasis, Esophageal squamous cell carcinoma

## Abstract

Krüpple-like factor 5 (KLF5) is a zinc-finger-containing transcription factor implicated in several human malignancies, but its potential regulatory mechanisms implicated in esophageal squamous cell carcinoma (ESCC) remain elusive. Here, we show that KLF5 is upregulated in ESCC, where its level was significantly associated with tumor differentiation and lymph node metastasis status. Upregulated KLF5 expression promoted the proliferation, migration, and invasion of ESCC cells. Reduced KLF5 showed the opposite effects. Mechanistically, KLF5 exerts its tumor promotion effect by up-regulating fibroblast growth factor binding protein 1 (FGF-BP1) and snail family transcriptional repressor 2 (SNAIL2). KLF5 binds to the promoter regions of FGF-BP1 and transcriptionally activates its expression. Our study indicated that KLF5 could promote esophageal squamous cell cancer proliferation, migration, and invasion by upregulating FGF-BP1/SNAIL2 signaling. Our work suggests that KLF5 might be a proto-oncogene in ESCC and implicated in ESCC metastasis.

## Introduction

Esophageal cancer (EsC) is a highly aggressive malignancy with an increasing prevalence in recent years. Esophageal squamous cell carcinoma (ESCC) and esophageal adenocarcinoma are the two main pathological types of EsC. It is estimated that ESCC type accounts for more than 90% of the total number of esophageal cancer cases in Asia, especially in China [1, 2]. Even with the remarkable progress in early diagnosis and advanced therapies, the outcomes for patients suffering from EsC remain poor, and 5-year survival rate is less than 14% [3]. For this reason, it is essential to identify new predictive markers and therapeutic targets to improve the survival of ESCC patients.

As a DNA-binding transcriptional regulator, the transcription factor Krüpple-like factor 5 (KLF5) can modulate the expression of several essential target genes, including cyclinD1 and surviving [4], regulate various biological processes, such as cell proliferation, growth, migration, and invasion. A large number of studies have implicated KLF5 as an oncogene in several cancer tissues, particularly in colorectal [5, 6], pancreatic [7], and breast cancer [8]. The high expression level of KLF5 has been reported to be an independent poor prognostic marker in breast cancer [9]. Knockdown of KLF5 significantly attenuates triple-negative breast cancer tumor growth [10]. In pancreatic ductal adenocarcinoma, KLF5 plays a crucial role in TGF-β-induced tumorigenesis [11]. Conversely, other studies describe KLF5 as a tumor suppressor. The KLF5 expression has been found to be downregulated in prostate cancer [12]. Recent studies show that KLF5 inhibits prostate cancer invasion through interactions with IGF1/STAT3 pathway [13].

A high expression level of KLF5 is observed in esophageal adenocarcinoma (EAC) cells and tissues. Reduced KLF5 in EAC cells has recently been shown to decrease cell proliferation and tumorigenicity [14]. KLF5 has also been reported to be lost in ESCC in a recent study, and overexpression of KLF5 in esophageal epithelia in vivo leads to promoting cell proliferation without squamous dysplasia or carcinoma [15]. Furthermore, our previous studies demonstrated that BAP1, a novel upstream positive regulator of KLF5 in breast cancer, contributes to ESCC cells proliferation and migration through enhancing KLF5 expression and its downstream genes, including Cyclin D1 and FGF-BP1 [16]. Given its pro-proliferative functions in several cell types, whether KLF5 is tumor-suppressive or oncogenic in ESCC remains debatable.

In the present study, we assessed the expression features of KLF5 in ESCC and determined upregulation of KLF5 expression was significantly associated with ESCC aggressiveness, such as poor differentiation and more lymph node metastasis. We confirmed that KLF5 overexpression promoted cell migration and invasion by upregulating the expression of FGF-BP1. Furthermore, we found that the FGF-BP1/SNAIL2 pathway involved the oncogenic effects of KLF5 in ESCC cells. Our results represent a new molecular mechanism by which KLF5 drives ESCC metastasis.

## Materials and methods

### Clinical samples and cell lines

Tumor specimens were obtained from ESCC patients at the time of surgery and were snap-frozen. The import and export standards for ESCC patients are as follows: Inclusion criteria: (1) All patients were diagnosed for the first time without a second primary tumor; (2) had not received anti-tumor therapy such as radiotherapy or systemic chemotherapy before or in the past; (3) Complete resection of lesions and dissection of lymph nodes during the operation; (4) Postoperative pathology was squamous cell carcinoma without other tumor components; (5) All specimens were preserved at the upper and lower incisal margins, and the pathologist confirmed that there was no tumor infiltration at the incisal margins. Exclusion criteria: (1) Incomplete resection of the tumor (R1, R2 resection); (2) Had received anti-tumor therapy such as systemic chemotherapy, radiation therapy or endoscopic resection before surgery; (3) Perioperative death caused by pulmonary embolism and massive hemorrhage during perioperative period. Medical Ethics Committee of the First Affiliated Hospital of Baotou Medical College approved the work. Verbal informed consent was obtained from the patients before the samples were collected and the experiments with human tissues conformed to the guidelines set by the Declaration of Helsinki. The cell lines ECa109 and Kyse150 were purchased from China Center for Type Culture Collection (Wuhan, China). Kyse140 cell line was a gift from Dr. Yan Li Sun from Yat-Sen University (Guangdong, China). ECa109, Kyse140, and Kyse150 were cultured in DMEM (Invitrogen, Carlsbad, CA, USA) supplemented with 10% fetal bovine serum (FBS, Gibco) and maintained in a humidified incubator with 5% CO_2_ at 37 °C.

### Immunohistochemistry staining

ESCC tumor tissues were subjected to immunohistochemistry staining (IHC) using anti-KLF5 rabbit polyclonal antibody (ab137676, 1:1000). Staining strictly followed the manufacturer’s protocol. IHC staining intensity was rated as ‘−’ (no staining), ‘+’ (light yellow), ‘++’ (light brown), and ‘+++’ (brown).

### Plasmids, small interfering RNA, and cell transfection

ECa109, Kyse140, and Kyse150 cells were seeded in 6-well plates and cultured to 70–80% confluency for transfection. The specific small interfering RNA targeting KLF5 (siKLF5), nonspecific control siRNA standard control (siNC), pcDNA-KLF5 plasmid, and blank control vector were transfected into the cells using Lipofectamine 2000 (Invitrogen, Carlsbad, CA, USA) according to the manufacturer’s instructions.

### Cell viability assays

The indicated number of cells was seeded into a 96-well plate, and the procedure was performed as described previously [17]. After transfected with siKLF5 or pcDNA-KLF5 plasmid in ESCC cells, cell viability was measured using the Cell Counting Kit-8 (CCK-8, Beyotime, China). The optical density of each well was quantified using a microplate reader.

### Wound-healing assay

The transfected ESCC cell suspensions were added into each well of 6-well cultured plates. A small pipette and a ruler were prepared for the vertical lines drawing. When the cells reached 70–80% confluency, they were wounded by scraping with the small pipette. After washing with phosphate-buffered saline (PBS) three times, the floating cells were removed, and 0.2 mL serum-free media was added. The scratched area was detected microscopically at 24 and 48 h after treatment and then further analyzed using ImageJ software.

### Transwell assays

For the transwell assays, the indicated ESCC cells in 0.1 mL serum-free medium were seeded into the upper chamber (Corning Costar), and 0.5 mL medium supplemented with 10% FBS was added into the lower chamber. After 24 h, the cells crossed the inserts, were fixed with 4% paraformaldehyde, and stained with crystal violet. After that, the invading cells on the basal side were counted under a Nikon optical microscope.

### Western blotting

KLF5, FGFBP1, and SNAIL2 expression were determined by western blotting analyses, which were performed as previously described [16]. The cells were lysed with RIPA buffer (Beyotime, China) for total protein extraction. Then, the protein concentration was calculated using a BCA assay Kit (Beyotime, China). Primary antibodies included in this study were listed as follows: KLF5 (ab137676, 1:1000, Abcam), FGFBP1 (ab215353, 1:500, Abcam), and SNAIL2 (EM1706-65, Cell Signaling Technology). Immunostaining was visualized with an ECL kit (Beyotime, China) and photographed.

### Bioinformatics analysis

Public RNA-sequencing data of ESCC patients from The Cancer Genome Atlas (TCGA, https://portal.gdc.cancer.gov/) and microarray data of ESCC patients from the Gene Expression Omnibus (GEO) (https://www.ncbi.nlm.nih.gov/gds/?term=, GSE16355, and GSE44021) were downloaded. Data of gene expression of ESCC tumors and adjacent noncancerous tissues were retrieved. A web server Gene Expression Profiling Interactive Analysis (GEPIA, http://gepia.cancer-pku.cn/index.html), an R language (https://www.r-project.org/) was used for gene expression analysis based on ESCC datasets from TCGA and GEO, respectively. The correlations between FGFBP1 and KLF5/SNAIL2 were explored using ESCC datasets from TCGA and GEO.

### Chromatin immunoprecipitation-polymerase chain reaction (ChIP-PCR)

For this test, a ChIP Kit from Thermo Fisher Scientific was purchased. In order to create DNA–protein cross-linking, treated cells were collected and fixed with 1% formaldehyde. After that, the cells were subjected to ultrasonic processing to create chromatin fragments. To immunoprecipitate the complex, cell lysate was then treated with the KLF5 antibody (rabbit, ab277773, Abcam). ChIP products were quantified using RT-qPCR. The primer sequences of the FGF-BP1 promoter were as follows: FGF-BP1-C1 forward: GCG GGG TGT TTG TGA GGA TA, and reverse: TGA CCA ATT TAC TCA GAG GCT CA; FGF-BP1-C2 forward: GTG TGA CAA TAC TGA GCC TAA TCC, and reverse: GCA ACT GAC TTT TGG GTT TGA GAT; FGF-BP1-C3 forward: AGA GCC TGG TAG AAT TCC CTG AT, and reverse: GGT GAC TGA AGT TGC CGA AGT; FGF-BP1-C4 forward: TCT CCA GAA CCA ATT CCT GCT TT, and reverse TGC CAA ACT ACT GAG CCC ATT.

### Dual-luciferase reporter assay

FGF-BP1 dual luciferase reporter gene vectors (H_FGF-BP1-WT) and mutant type (MUT) (H_FGF-BP1-MT) plasmids with KLF5 binding sites (FGF-BP1-C1) were constructed respectively. The H-KLF5 and pcDNA3.1(+) plasmids and the reporter plasmids were then co-transfected into ECa109 cells. Dual-Luciferase® Reporter Assay System (Promega Corporation, Madison, WI) was used to measure the luciferase activity after 48 h.

### Statistical analysis

The measurement data were expressed with mean ± standard deviation (SD). The relative expression level of KLF5 and the number of invaded cells were plotted using GraphPad Prism 5 software (GraphPad Software, San Diego, CA, USA). All statistical analyses were performed in SPSS 20.0 (IBM, Armonk, NY, USA). A statistical difference was determined for *P* < 0.05.

## Results

### Upregulation of KLF5 was associated with ESCC aggressiveness

**Fig. 1:**
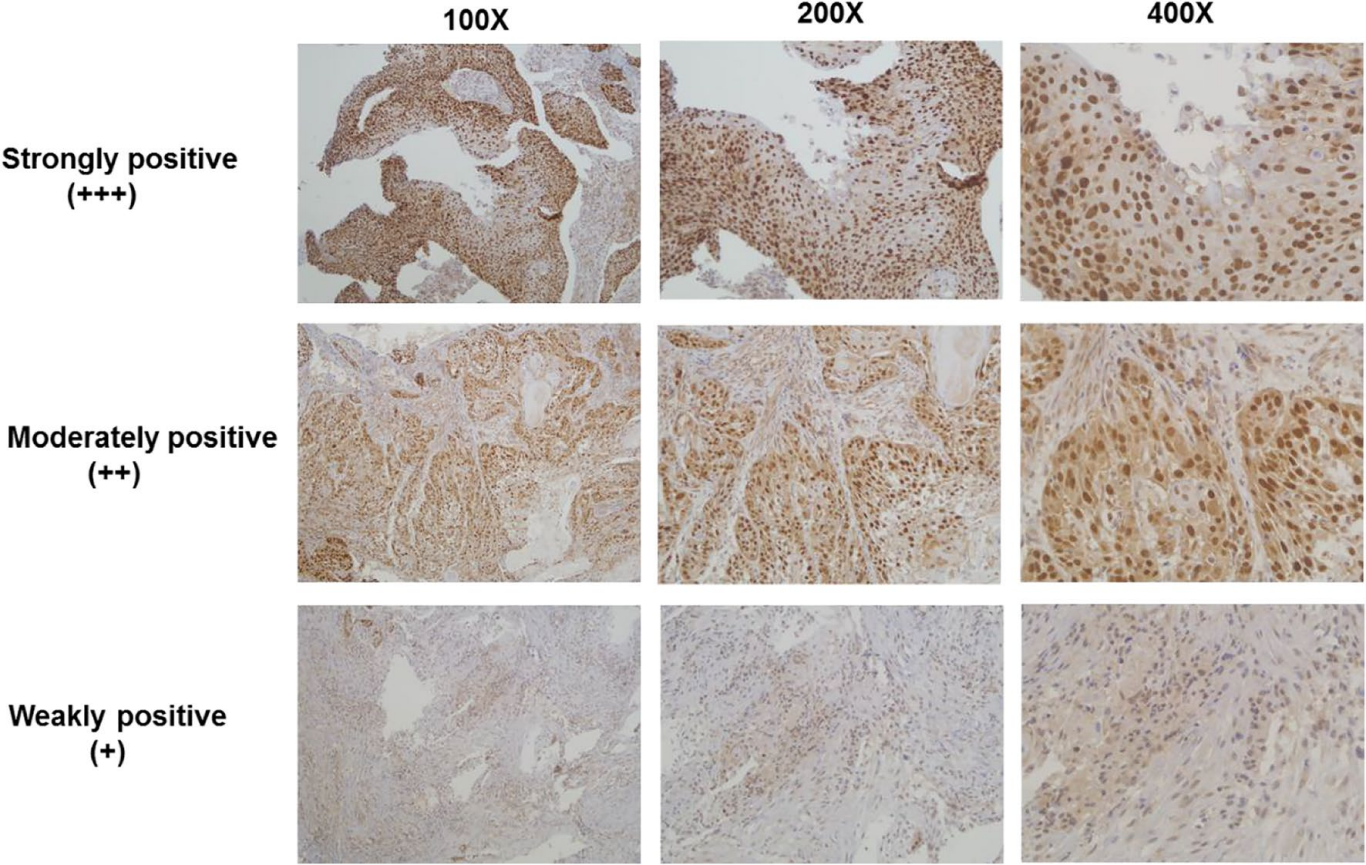
Representative pictures of immunohistochemistry staining for KLF5 in ESCC tissues

Among the 47 ESCC samples, the protein expression of KLF5 was detected in 37 ESCC tissues by IHC. The staining was observed in the cellular cytoplasm and nucleus (Fig. 1). Twenty-one samples (44.7%) had no or little KLF5 expression (low group, scores 0, 1+), whereas 26 patients (55.3%) exhibited strong staining (high group, scores 2+, 3+). Next, the correlations between the expression level of KLF5 and the clinicopathological features of ESCC were investigated. We found that up-regulation of KLF5 expression was significantly associated with ESCC aggressiveness, such as poor differentiation (*P* = 0.041) and more lymph node metastasis (*P* = 0.037) (Table 1).

**Table 1:** Relationship between KLF5 Expression and clinicopathologic characteristics in ESCC patients

Variables	Case *n* (%)	KLF5 Expression (%)			
		Low (*n* = 21)	High (*n* = 26)	*p*-value	*χ*^*2*^
Age (years)				0.970	0.001
≤ 60	20	9 (45%)	11 (55.0%)		
≥ 60	27	12 (44.4%)	15 (55.6%)		
Sex				0.529	0.396
Male	29	14 (48.3%)	15(51.7%)		
Female	18	7 (38.9%)	11 (61.1%)		
Tumor location				0.621	0.954
Upper	10	5 (50.0%)	5 (50.0%)		
Middle	26	10 (38.5%)	16 (61.5%)		
Lower	11	6 (54.5%)	5 (45.5%)		
Histologic grade				0.134	4.023
G1	13	8 (61.5%)	5 (38.5%)		
G2	21	10 (47.6%)	11 (52.4%)		
G3	13	3 (23.1%)	10 (76.9%)		
Pathologic differentiation				0.0012	10.49
Poor	9	2 (22.2%)	7 (77.8%)		
Moderate	24	8 (37.5%)	16 (62.5%)		
Well	14	12 (71.4%)	2 (28.6%)		
Lymph node metastasis				0.037	4.362
Yes	14	3 (21.4%)	11 (78.6%)		
No	33	18 (54.5%)	15 (45.5%)		

### KLF5 promoted ESCC cells proliferation

**Fig. 2:**
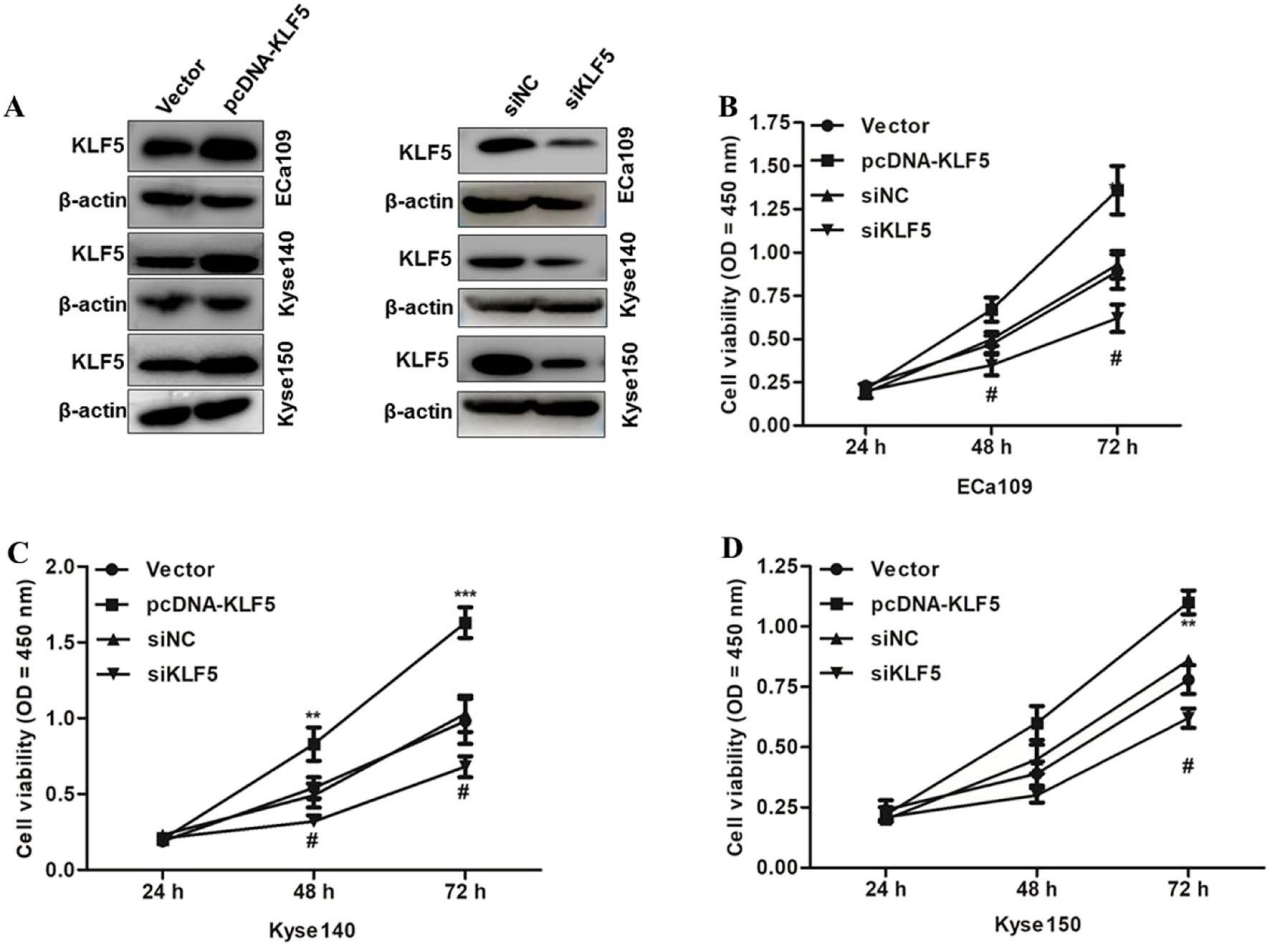
The effect of KLF5 overexpression/knockdown on ESCC proliferation. **A** The protein expressions of KLF5 in Eca109, Kyse140, and Kyse150 cells that have been transfected with empty control, pcDNA-KLF5 vector or siNC, siKLF5 vector were determined by western blotting. **B** CCK-8 assay for Eca109 that have been transfected with empty vector, pcDNA-KLF5, siNC, and siKLF5. **C** CCK-8 assay for Kyse140 that have been transfected with empty vector, pcDNA-KLF5, siNC, and siKLF5. **D** CCK-8 assay for Kyse150 that have been transfected with empty vector, pcDNA-KLF5, siNC, and siKLF5. *Indicated the difference in cell variability between empty control and pcDNA-KLF5 group, and ^#^indicated the difference in cell variability between siNC and siKLF5 group. *P < 0.05, **P < 0.01, ***P < 0.001, ^#^*P* < 0.05

To investigate the biological roles of KLF5 in ESCC tumorigenesis, CCK-8 assays were performed to evaluate the effects of KLF5 overexpression/knockdown on ESCC cell proliferation. In the present work, KLF5 was overexpressed in Eca109, Kyse140, and Kyse150 after plasmid pcDNA-KLF5 transfection. In addition, ESCC cell lines presented the knockdown of KLF5 when plasmid siKLF5 was transfected (Fig. 2A). Cell proliferation was more vital in the pcDNA-KLF5 group in three ESCC cell lines than that in the blank control group at 48- and 72-h post-transfection (*P* < 0.05), while cell proliferation was significantly weaker in the siKLF5 group in three ESCC cell lines than that in the siNC at 48- or 72-h post-transfection (*P* < 0.05; Fig. 2B, C and D). KLF5 overexpression promoted ESCC cell proliferation; in contrast, KLF5 loss inhibited ESCC cell proliferation.

### KLF5 overexpression promoted the migration and invasion of ESCC cells in vitro

**Fig. 3:**
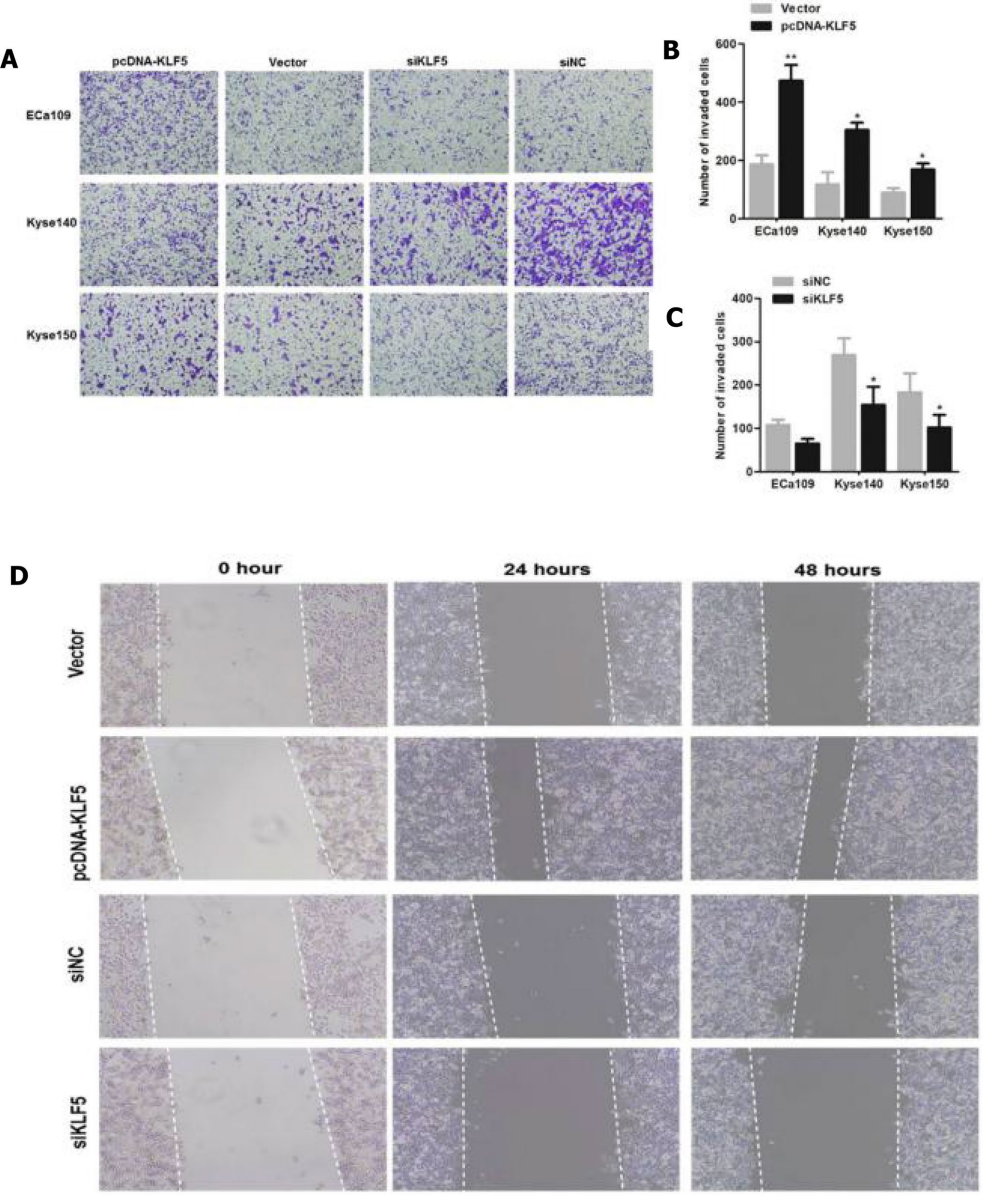
Transwell assay for Eca109, Kyse140, and Kyse150 cells transfected with empty vector, pcDNA-KLF5 or siNC, siKLF5 were evaluated at 24 h after transfection. **A**–**C** Representative picture of the transwell assay in ESCC cells at 24 h after transfection. KLF5 overexpression in ESCC cells promoted cell invasion; while silencing of KLF5 inhibited cell invasion. **D** Wound healing assay for Eca109 cells transfected with empty vector, pcDNA-KLF5, siNC, and siKLF5. *Indicated the difference in cell variability between control and pcDNA-KLF5 group or siKLF5 group. *P < 0.05, **P < 0.01

Cell migration and invasion are critical biological processes contributing to tumor metastasis. Next, we determined whether KLF5 was involved in the metastasis of ESCC. Transwell invasion assay indicated that KLF5-overexpressed Eca109, Kyse 140, and Kyse 150 cells exhibited significantly increased invasion capabilities compared with their corresponding control cells, while KLF5-reduced Kyse140 and Kyse150 cells revealed significantly decreased invasion capabilities compared with their corresponding control cells at 24-h after transfection (*P* < 0.05, Fig. 3A, B, C). Meanwhile, the results of the wound healing assay showed that the scratch in KLF5-overexpressed Eca109 cells was smaller than that of in control cells both at 24 and 48 h after transfection, while the scratch in KLF5-reduced Eca109 cells was larger than that of in control cells at 48-h after transfection (Fig. 3D). These findings demonstrated that KLF5 overexpression promoted ESCC cell migration and invasion.

### FGF-BP1 is a transcriptional target of KLF5

**Fig. 4:**
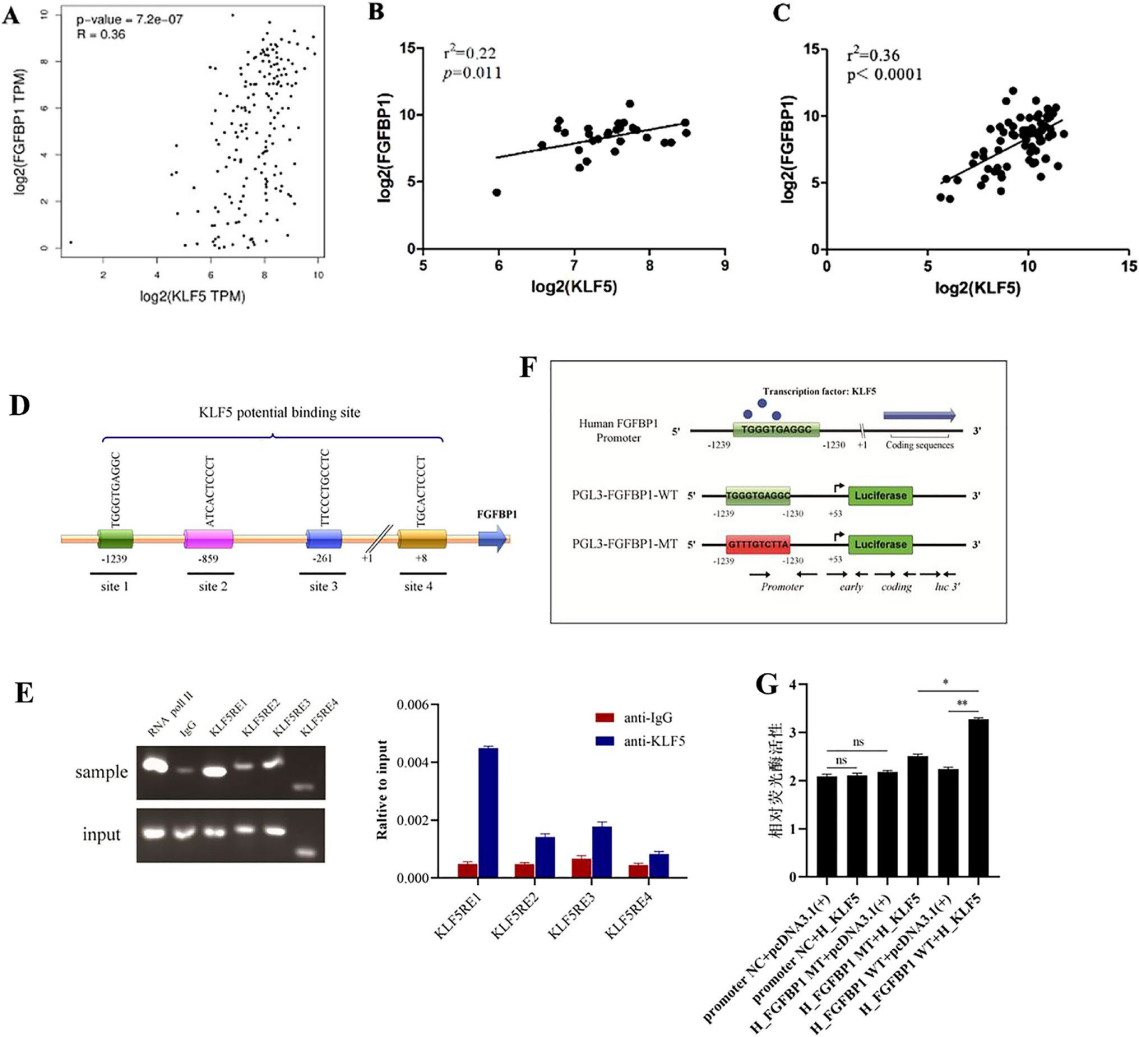
FGF-BP1 is a transcriptional target of KLF5 in ESCC. **A** The correlations of mRNA expression level between KLF5 and FGF-BP1 based on TCGA dataset; **B** the correlations of mRNA expression level between KLF5 and FGF-BP1 based on GEO16355 dataset; **C** the correlations of mRNA expression level between KLF5 and FGF-BP1 based on GEO44021 dataset; **D** binding motif for KLF5 was predicted by JASPAR database; **E** ChIP-PCR assay revealed direct binding of KLF5 to the promoter region of FGF-BP1; **F** and **G** dual luciferase reporter assay confirmed that KLF5 significantly increased FGF-BP1 promoter activity. *P < 0.05

The above results from Fig. 3 suggested the involvement of KLF5 in ESCC cell metastasis. Next, the TCGA database was processed to explore the possible mechanism underlying KLF5-driving ESCC malignancy. TCGA analysis revealed a significantly positive correlation between KLF5 and FGF-BP1 mRNA expression in ESCC specimens (Fig. 4A). To confirm the interaction between KLF5 and FGF-BP1, two human microarray datasets (GSE16355 and GSE44021) from GEO were used to evaluate the mRNA expression of KLF5 and FGF-BP1. Likewise, the positive correlation between KLF5 and FGF-BP1 was also found in GEO datasets (Fig. 4B and C). These findings indicated that FGF-BP1 might be implicated in the biological functions of KLF5 in ESCC.

**Fig. 5:**
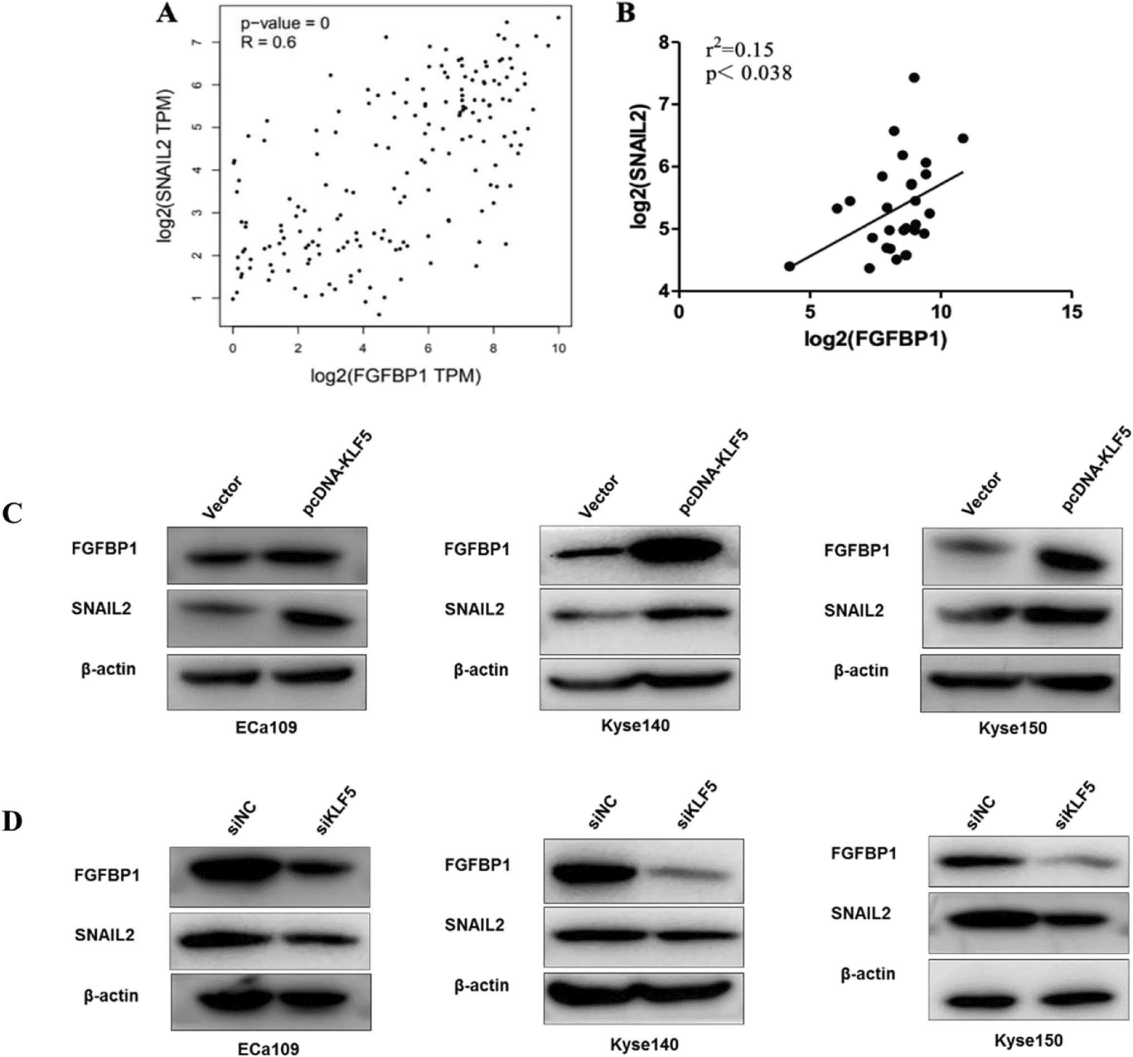
KLF5 regulates the expression of FGF-BP1 and SNAIL2. **A** The correlations of mRNA expression level between SNAIL2 and FGF-BP1 based on TCGA dataset. **B** The correlations of mRNA expression level between SNAIL2 and FGF-BP1 based on GEO16355 and GEO44021 datasets. **C** The expression of FGF-BP1 and SNAIL2 in Eca109, Kyse140, and Kyse150 cells transfected with empty vector and pcDNA-KLF5; **D** The expression of FGF-BP1 and SNAIL2 in Eca109, Kyse140, and Kyse150 cells transfected with siNC and siKLF5

We proposed that KLF5 may directly encourage the transcription of FGF-BP1 since it acts as a transcription factor. The JASPAR database was used to predict the potential binding sites between KLF5 and FGF-BP1 promoters. According to the prediction of JASPAR, there were four potential binding sites for KLF5 in the FGF-BP1 promoter region (Fig. 4D, − 1239, − 859, − 261, + 8). ChIP-PCR assay showed that the KLF5 could only bind to the promoter region of FGF-BP1 at − 1239 bp (FGF-BP1-C1) upstream from the start codon (Fig. 5E). Dual-luciferase reporter assay further showed that KLF5 promoted the luciferase activity of FGF-BP1-WT, with no significant effects on the luciferase activity of FGF-BP1-MT ((Fig. 4F and G). These data indicated that FGF-BP1 is a transcriptional target of KLF5 in ESCC.

### KLF5 promoted ESCC invasion via activating EMT through FGF-BP1/SNAIL2 signaling

Generally, epithelial-mesenchymal transition (EMT) activation is critical to cancer migration and invasion. FGF-BP1 signaling has been reported to be involved in EMT, migration, and invasion in several cancers [18, 19]. Here, we investigated the interaction between FGF-BP1 and EMT-related genes using the web server GEPIA and GEO datasets (GSE16355). Notably, the positive correlation between FGF-BP1 and EMT-related genes SNAIL2 was revealed (Fig. 5A and B). Based on these findings, we hypothesized that the involvement of KLF5 in ESCC metastasis was associated with EMT through the FGF-BP1 pathway by regulating SNAIL2 expression. To preliminarily validate our hypothesis, we performed western blots to assess the protein expression of FGF-BP1 and SNAIL2 in ESCC cells following KLF5 knockdown or overexpression. As shown in Fig. 5C, KLF5 overexpression induced the apparent increase of FGF-BP1 and SNAIL2 protein abundances in ESCC cells. As expected, the opposite expression pattern of FGF-BP1 and SNAIL2 was verified in ESCC cells with KLF5 knockdown (Fig. 5D). Thus, KLF5 exerts its function on ESCC invasion, and metastasis via FGF-BP1/SNAIL2 mediated EMT process.

## Discussion

ESCC is one of the most prevalent malignant tumors in Asian countries. Although the mortality rate of ESCC appears to have decreased in the last decades, it still ranks fourth in all forms of malignant tumors [20]. Due to the lack of obvious symptoms and sensitive tumor biomarkers, many ESCC patients missed opportunities for effective surgery at the time of diagnosis [21]. It is even more challenging that about half of patients are present with distant metastasis at initial diagnosis and one-third develop local recurrences after surgery [22]. Tumor invasion and metastasis were the leading causes of ESCC-induced death. Therefore, uncovering the molecular mechanisms in cancer invasion or metastasis paves the way for early diagnosis and the development of targeted therapy, thus improving the clinical outcomes of ESCC patients.

KLF5 gene is located at chromosome region 13q21 and contains four exons spanning 18.5 kb. Although evidence has demonstrated the critical functions of KLF5 in pathological processes of tumor progression [23], whether KLF5 is a tumor suppressor or oncogene is still debatable. In the present study, we demonstrated the upregulated protein expression of KLF5 in ESCC tissues. Moreover, the elevated KLF5 level was closely related to aggressive tumor phenotypes, including poor differentiation and distant metastases [24]. For function analysis, in vitro experiments were designed to clarify the potential role of KLF5 in the progression of ESCC. As expected, KLF5 promoted cell proliferation, migration, and invasion. These findings strongly suggest that KLF5 might act as an oncogene in ESCC.

The current study also determined the molecular mechanisms by which KLF5 is involved in ESCC metastasis. Using public databases in GEO and TCGA, we found that the mRNA expression level of KLF5 positively correlated with that of FGF-BP1, which could bind to fibroblast growth factors such as FGF2, thus promoting the progression of cancer disease, including ESCC. Western blot analysis confirmed the increased protein expression level of FGF-BP1 following KLF5 overexpression. Our research also revealed that KLF5 is bound to the FGF-BP1 promoter, increasing the promoter's transcriptional activity as a consequence. These findings revealed that KLF5 might promote tumor progression of ESCC by activating the FGF-BP1 signaling pathway.

EMT program is a necessary cell-biological process for cancer invasion, thus contributing to metastasis phenotype [25]. SNAIL2, as an essential EMT inducer, is known to regulate several epithelial-specific genes and thereby modulate the migration and invasion process. In ESCC, SNAIL2 was reported to promote EMT activation and distant metastasis [26, 27]. Using GEO datasets and TCGA data, we found that the mRNA expression level of FGF-BP1 had a significantly positive correlation with that of SNAIL2. Subsequently, Western blot analysis confirmed that enforced expression of KLF5 up-regulated the expression of SNAIL2. We suspect that KLF5 can interact with FGF-BP1 to promote ESCC metastasis by inducing the EMT program via the FGF-BP1/SNAIL2 axis.

Several limitations of our study should be considered. First, whether FGF-BP1 could directly target SNAIL2 was not determined. Second, in vivo experiments should be performed to validate the roles of KLF5 in cancer proliferation, migration, and invasion. Therefore, our future research will primarily aim to elucidate the specific roles of FGF-BP1 and SNAIL2, as well as confirm the involvement of KLF5 in the progression of ESCC through in vivo experiments.

## Conclusion

Collectively, the key findings of this study demonstrated that KLF5 promoted tumor proliferation and metastasis in ESCC cells. Furthermore, we emphasized the regulatory mechanisms by which KLF5 promoted invasion and metastasis of ESCC via activating the EMT program by regulating FGF-BP1/SNAIL2. These results preliminarily indicated that KLF5 might be an oncogene in ESCC and implicated in ESCC metastasis.

## Data Availability

The data generated in the present study are included in the figures and/or tables of this article.
